# Prevention of Respiratory Infections in Children with Congenital Heart Disease: Current Evidence and Clinical Strategies

**DOI:** 10.3390/vaccines14010011

**Published:** 2025-12-22

**Authors:** Susanna Esposito, Camilla Aurelio, Marina Cifaldi, Angela Lazzara, Federico Viafora, Nicola Principi

**Affiliations:** 1 Pediatric Clinic, Department of Medicine and Surgery, University of Parma, 43126 Parma, Italy; camilla.aurelio@unipr.it (C.A.); marina.cifaldi@unipr.it (M.C.); angelamaria.lazzara@unipr.it (A.L.); federico.viafora@unipr.it (F.V.); 2 Università degli Studi di Milano, 20122 Milan, Italy; nicola.principi@unimi.it

**Keywords:** congenital heart disease, respiratory infections, vaccination, monoclonal antibodies, respiratory syncytial virus, influenza, SARS-CoV-2, pneumococcal disease

## Abstract

*Background:* Children with congenital heart disease (CHD) are at substantially increased risk for respiratory infections, which occur more frequently and with greater severity than in healthy peers. This heightened vulnerability stems from multifactorial immune impairment, including defects in innate and adaptive immunity, chronic inflammation related to abnormal hemodynamics and hypoxia, reduced thymic function, and genetic syndromes affecting both cardiac and immune development. Viral pathogens—particularly respiratory syncytial virus (RSV), influenza viruses, and SARS-CoV-2—account for most infections, although bacterial pathogens remain relevant, especially in postoperative settings. *Methods:* This narrative review summarizes current evidence on infection susceptibility in children with CHD, the epidemiology and clinical relevance of major respiratory pathogens, and the effectiveness of available preventive measures. Literature evaluating immunological mechanisms, infection burden, vaccine effectiveness, and passive immunization strategies was examined, along with existing national and international immunization guidelines. *Results:* Children with CHD consistently exhibit higher rates of hospitalization, intensive care unit admission, mechanical ventilation, and mortality following respiratory infections. RSV, influenza, and SARS-CoV-2 infections are particularly severe in this population, while bacterial infections, though less common, contribute substantially to postoperative morbidity. Preventive options—including routine childhood vaccines, pneumococcal and *Haemophilus influenzae* type b vaccines, influenza vaccines, COVID-19 mRNA vaccines, and RSV monoclonal antibodies—demonstrate strong protective effects. New long-acting RSV monoclonal antibodies and maternal vaccination markedly enhance prevention in early infancy. However, vaccine coverage remains insufficient due to parental hesitancy, provider uncertainty, delayed immunization, and limited CHD-specific evidence. *Conclusions:* Respiratory infections pose a significant and preventable health burden in children with CHD. Enhancing the use of both active and passive immunization is essential to reduce morbidity and mortality. Strengthening evidence-based guidelines, improving coordination between specialists and primary care providers, integrating immunization checks into routine CHD management, and providing clear, condition-specific counseling to families can substantially improve vaccine uptake and clinical outcomes in this vulnerable population.

## 1. Introduction

Congenital heart diseases (CHDs) encompass structural abnormalities of the heart or great vessels present at birth and represent the most common congenital anomaly worldwide, affecting more than 15 million children—approximately 1% of the population [1]. CHDs account for 3% of neonatal deaths and nearly half of all deaths attributed to congenital malformations [2]. When these defects significantly impair blood flow, they are classified as hemodynamically significant CHDs (HSCHD). Among them, the subset requiring urgent medical or surgical intervention in early infancy is defined as critical CHD (CCHD). The most frequent CCHDs include Tetralogy of Fallot, Transposition of the Great Arteries (TGA), Hypoplastic Left Heart Syndrome, Pulmonary Atresia, Total Anomalous Pulmonary Venous Return, Tricuspid Atresia, and Truncus Arteriosus [3].

Children with CHD face substantially higher risks of both cardiac complications and systemic health problems compared with their healthy peers. Cardiovascular issues such as heart failure, stroke, atrial fibrillation, hypercyanotic spells, and severe pulmonary hypertension are common [4], and growth failure affects nearly half of affected children [5]. Infections, however, represent the most frequent and burdensome complications. Premature infants with CHD are particularly vulnerable to sepsis, with up to 35% of late-onset sepsis cases occurring in the presence of a patent ductus arteriosus [6]. Respiratory infections are especially prevalent: in a multicenter Italian study of 420 children with HSCHD, 63.1% experienced at least one respiratory illness within the first two years of life, most commonly upper respiratory tract infections (76.4%), acute bronchitis (43.3%), and influenza (22.1%) [7]. Similarly, a review of nearly 29,000 hospital discharges found respiratory infections in 26% of children with CHD, with the most severe outcomes occurring in those with critical defects [8]. Both studies showed that respiratory infections in CHD significantly increase intensive care unit (ICU) admission rates, length of hospitalization, mortality risk, and impose considerable social and economic burdens.

Preventing respiratory infections in children with CHD is therefore essential, yet remains challenging. General infection-control measures often represent the only available strategies, and vaccination coverage remains suboptimal despite clear recommendations. Progress in passive immunization has been slow, and earlier products have not always provided sufficient protection. Nevertheless, recent developments—including improved vaccines and monoclonal antibodies—have expanded opportunities for prevention. This narrative review was conducted to summarize current knowledge on the burden, causes, and prevention of respiratory infections in children with CHD, with a focus on vaccines and monoclonal antibody–based strategies.

## 2. Methods

This review followed established principles for narrative biomedical reviews and incorporated peer-reviewed research, epidemiological data, and guidance from major health organizations.

A comprehensive literature search was performed in PubMed/MEDLINE, Scopus, and Web of Science for studies published from 1969 through 2025. Search terms included combinations describing congenital and critical heart defects, respiratory infections and their common viral and bacterial causes, and preventive interventions such as vaccination and monoclonal antibodies. Additional articles were identified by manually reviewing relevant bibliographies and consulting current immunization and disease-prevention guidelines issued by international health authorities.

Studies were eligible for inclusion if they involved pediatric populations with CHD and reported information on immune function, incidence or severity of respiratory infections, or preventive strategies such as vaccination, passive immunization, or infection-control measures. The review considered original research studies, systematic reviews, narrative reviews, and clinical or public health guidelines. Studies were excluded if they did not involve children, did not address respiratory outcomes, were unavailable in English, or consisted only of isolated case reports or very small case series unless they provided essential clinical insights.

The literature identification, selection, and synthesis were conducted collaboratively by all authors. Following the initial database searches, retrieved records were screened independently by multiple authors (CA, MC, AL, and FV) based on predefined inclusion and exclusion criteria. Discrepancies regarding eligibility were resolved through discussion and consensus, with senior authors (SE and NP) acting as arbiters when needed. The quality and strength of the evidence were assessed narratively rather than through formal scoring systems, taking into account study design, sample size, clarity of CHD definition, methodological rigor, consistency of findings, and relevance to current clinical practice. Evidence from randomized controlled trials, large observational studies, and authoritative guidelines was weighted more heavily than small case series or anecdotal reports. Thematic areas highlighted in the review were identified through an iterative process: key findings emerging from the literature were grouped into preliminary thematic domains, which were then refined through repeated team discussions to ensure coherence, clinical relevance, and comprehensive coverage of the field. This consensus-driven approach allowed integration of heterogeneous evidence into a structured narrative framework.

Data were extracted on study design, population characteristics, classification of CHD, infectious agents studied, and reported clinical outcomes such as hospitalization, intensive care admission, or mortality. For preventive measures, additional information was collected regarding immunization schedules, protective efficacy, safety, and regulatory status. Because of the heterogeneity of study designs, populations, and outcomes, a formal meta-analysis was not performed. Instead, results were synthesized qualitatively and organized into thematic areas addressing susceptibility to infection, the epidemiology of major pathogens, and available preventive interventions.

Public health guidelines were evaluated based on the expertise of the issuing organization and the recency of publication.

The uploaded manuscript served as an initial guide for identifying central themes, but the literature search and analysis were conducted independently to ensure comprehensive and unbiased coverage.

## 3. Reasons for the Increased Susceptibility to Infection of Children with Congenital Heart Disease

Children with CHD exhibit a markedly increased susceptibility to infections, primarily due to impairments across multiple components of the immune system. Numerous studies have demonstrated reduced neutrophil effector function and diminished bacterial killing capacity in these patients [9]. In addition, many children with CHD present with decreased numbers of T and B lymphocytes and reduced production of naïve T cells [10]. Lower concentrations of T-cell Receptor Excision Circles (TRECs)—a marker of thymic output—have also been reported [11]. Serum levels of IgA, IgG, and complement components are frequently diminished, while suppressor T-cell function may be increased [12]. These immune abnormalities tend to be more frequent and more severe in children with CCHD, cyanotic defects, and conotruncal malformations compared with those who have shunt lesions or obstructive defects [13].

Several mechanisms contribute to these immune disturbances. Chronic inflammation represents one of the major underlying factors. Structural heart defects can lead to abnormal hemodynamics and cardiac remodeling, which in turn generate mechanical stress and trigger persistent production of inflammatory and apoptotic mediators [14]. Elevated concentrations of cytokines such as IL-6, IL-10, and TNF-α have been documented in children with coarctation of the aorta [15], while increased acute-phase reactants and interferon-γ levels have been observed in those with ventricular septal defects [16]. Intestinal dysbiosis further amplifies systemic inflammation: chronic hypoxia, common in many severe CHDs, alters the composition and function of the gut microbiota [17], enabling microbial products to translocate into the bloodstream and stimulate inflammatory pathways [18]. Recurrent infections may then perpetuate and intensify this chronic inflammatory state [19].

Hypoxia itself is an additional contributor to immune dysfunction. In cyanotic CHD, reduced oxygen availability impairs the generation of reactive oxygen species (ROS) and inhibits the formation of neutrophil extracellular traps (NETs), thereby weakening the capacity of neutrophils to neutralize pathogens [20,21].

Impaired thymic function also plays a critical role. The thymus is essential for T-cell maturation, and its early removal or damage can have long-term consequences for adaptive immunity. During corrective cardiac surgery in infancy, partial or complete thymectomy is sometimes necessary to access the heart. A systematic review of 21 studies found that early thymectomy significantly reduces the numbers of total, CD4+, CD8+, and naïve T cells, along with decreases in TRECs, CD31 expression, and T-cell receptor repertoire diversity, particularly during the first five years of life [22]. Even in the absence of surgery, thymic atrophy has been observed in some infants with complex CHD. In a study of 58 infants evaluated prior to cardiac surgery, those with complex or cyanotic defects showed substantial loss of double-positive thymocytes, leading to reduced thymic output and impaired T-cell repertoire formation [14,15,16,17,18,19,23].

Genetic conditions that include both CHD and immune abnormalities provide additional evidence for a shared biological basis underlying infection susceptibility. Several syndromes—most notably 22q11.2 deletion syndrome, Down syndrome (DS), and Turner syndrome (TS)—combine cardiac malformations with intrinsic immune deficiencies. In 22q11.2 deletion syndrome, impaired development of the third and fourth pharyngeal arches results in abnormalities of the cardiac outflow tract, thymus, and parathyroid glands; affected children commonly present with tetralogy of Fallot, truncus arteriosus, interrupted aortic arch, or ventricular septal defects, accompanied by thymic insufficiency and T-cell deficits [24]. In DS, trisomy 21 leads to wide-ranging abnormalities in both innate and adaptive immunity, including defects in T-cell and B-cell maturation, impaired neutrophil and monocyte function, and features of immune dysregulation [25]. Approximately half of children with DS have CHD—most often atrioventricular septal defects, ventricular or atrial septal defects, patent ductus arteriosus, or tetralogy of Fallot [26]. Overexpression of interferon receptor genes on chromosome 21 may disrupt cardiac development by altering Wnt signaling [27,28], and increased interferon-stimulated gene activity is thought to contribute to a state of chronic, mild interferonopathy associated with increased infection risk [29]. Turner syndrome, characterized by partial or complete monosomy X, is also associated with congenital cardiac anomalies, particularly bicuspid aortic valve and coarctation of the aorta [30], as well as immune deficiencies such as reduced T follicular helper cells and impaired antibody generation [31].

Collectively, these immune, inflammatory, hypoxic, and genetic factors interact to create a biological environment in which children with CHD are substantially more vulnerable to infections than their healthy peers.

Table 1 summarizes the immune mechanisms underlying increased infection susceptibility in children with CHD.

## 4. Etiology and Clinical Relevance of Respiratory Infections in Children with Congenital Heart Disease

Most respiratory infections in children with CHD are viral, mirroring patterns observed in otherwise healthy pediatric populations. Common viral pathogens include respiratory syncytial virus (RSV), influenza viruses, metapneumovirus, adenovirus, and, more recently, SARS-CoV-2. However, both the incidence and severity of these infections are substantially greater in children with CHD. Although bacterial infections occur less frequently, they can lead to severe complications, particularly following cardiac surgery. The major viral and bacterial agents associated with morbidity in this population are described below.

In addition to pathogen-specific preventive strategies, strict adherence to routine childhood immunization schedules remains essential for children with CHD. Vaccines against measles and pertussis are particularly important, as both infections can cause severe respiratory illness, hypoxemia, and systemic complications that may precipitate cardiac decompensation in vulnerable patients. Although large outbreaks of measles and pertussis are currently less frequent in many high-income countries due to widespread vaccination, periodic resurgences continue to occur, especially in settings with declining vaccine coverage. Children with CHD may therefore be at increased risk of adverse outcomes if infected. Ensuring timely administration of measles-containing vaccines and pertussis-containing vaccines, according to national and international recommendations, represents a fundamental component of comprehensive infection prevention in this population.

### 4.1. Viral Infections

#### 4.1.1. Respiratory Syncytial Virus

RSV is one of the leading causes of lower respiratory tract infections (LRTIs) worldwide, responsible for more than 3.6 million hospitalizations and around 100,000 deaths annually in children under five years of age. Its clinical impact on children with CHD has been recognized for decades. Early data showed that infants with CHD experienced markedly more severe RSV bronchiolitis than those without CHD, including higher rates of ICU admission (63% vs. 14%), increased need for mechanical ventilation (22% vs. 5%), and substantially elevated mortality (37% vs. 1.5%) [32]. Subsequent studies have consistently confirmed that children with complex, hemodynamically significant, or critical CHD carry the highest risk of severe RSV disease. RSV infection can also adversely affect postoperative outcomes, particularly when cardiac surgery occurs during early infancy [33,34,35,36].

A comprehensive systematic review and meta-analysis by Chaw et al. [37] further highlighted the disproportionate burden of RSV in CHD. Beyond confirming more severe disease and a strong correlation between infection severity and CHD complexity, the authors noted that severe RSV outcomes frequently occurred in children older than one year of age. In one included study, the median age of RSV hospitalization was 5.7 months (range 2.1–19.4) in children with CHD versus 4.3 months (range 1.1–23.6) in those without CHD [38]. Another study reported median ages of 7.0 months (range 0–23) versus 6.0 months (range 0–23) for CHD and non-CHD children, respectively [39]. These findings indicate that effective RSV prevention strategies should extend well beyond the early months of life, especially in CHD patients.

#### 4.1.2. Influenza Viruses

Influenza viruses cause approximately 90 million infections each year among children under five, with a considerable proportion progressing to severe disease requiring hospitalization and occasionally leading to death [40]. Children with chronic health conditions, including CHD, are at particularly high risk for severe influenza-related complications [41].

Evidence from the U.S. Kids’ Inpatient Database demonstrated that children with CHD hospitalized for influenza had significantly worse outcomes than their peers without CHD [42]. Among 125,470 influenza-related admissions, the 2174 children with CHD had higher odds of mortality (aOR 2.8), acute respiratory failure (aOR 1.8), acute kidney injury (aOR 2.2), mechanical ventilation (aOR 1.9), and prolonged hospitalization (median 4 vs. 2 days). Notably, CHD severity did not affect the magnitude of these risks.

A second large study using the Pediatric Health Information System (PHIS) database confirmed these findings [43]. Among more than 55,000 hospitalized children, those with CHD again had higher mortality (4.1% vs. 0.9%), increased likelihood of requiring mechanical ventilation, higher ICU admission rates, and longer hospital stays. These data underscore influenza as a major cause of preventable morbidity in children with CHD.

#### 4.1.3. SARS-CoV-2

Although SARS-CoV-2 infection is mild in most healthy children, approximately 3–6% develop severe disease requiring hospitalization, with younger children at greatest risk [44,45]. This risk is significantly amplified in children with serious underlying conditions such as CHD [46,47,48,49].

In comparative analyses, Ghimire et al. [42] reported no significant difference in mortality between children with and without CHD (1.2% vs. 0.8%), but children with CHD experienced markedly higher rates of tachyarrhythmias (aOR 4.2), heart block (aOR 5.0), respiratory failure (aOR 2.0), and acute kidney injury (aOR 3.4). They also required noninvasive and invasive mechanical ventilation more often and had longer hospital stays.

A recent multicenter study corroborated these findings [50], showing that while CHD did not increase COVID-19 mortality, it significantly elevated the risks of arrhythmias, need for ventilation, and acute kidney injury. Children under three years accounted for the majority of critical cases, and those with complex CHD anatomy had a 4.3-fold higher risk of ICU admission.

Table 2 shows the burden and clinical impact of key respiratory viruses in children with CHD.

### 4.2. Bacterial Infections

Although less frequent than viral illnesses, bacterial LRTIs represent an important threat to children with CHD. The most common pathogens—*Streptococcus pneumoniae*, *Haemophilus influenzae*, and *Mycoplasma pneumoniae*—mirror those affecting healthy children. However, children with CHD have a significantly higher risk of hospitalization for *H. influenzae* pneumonia (aOR 2.1), while the risk for *S. pneumoniae* pneumonia appears slightly reduced (aOR 0.9) compared with the general pediatric population [51]. Regardless of bacterial cause, CHD is associated with higher mortality, longer hospital stays, and increased rates of complications such as respiratory failure, acute kidney injury, and cardiac arrest.

Nosocomial bacterial infections are particularly relevant after cardiac surgery. In a cross-sectional study of 135 infants undergoing open-heart procedures, postoperative infection occurred in 11.96% of patients, with *Acinetobacter*, *Pseudomonas aeruginosa*, and *Enterobacter* spp. among the most frequently isolated Gram-negative organisms, and *Corynebacterium diphtheriae* and *Staphylococcus epidermidis* among Gram-positive pathogens. *Candida albicans* was also commonly isolated [52]. A second study involving 300 children requiring cardiac surgery reported an even higher postoperative infection rate of 40%, with *S. epidermidis*, *Staphylococcus aureus, Enterococcus*, *P. aeruginosa*, and *C. albicans* as the predominant pathogens [52].

## 5. Prevention of Respiratory Infections in Children with Congenital Heart Disease

Because children with CHD are at substantially increased risk of respiratory infections and their complications, rigorous prevention strategies are essential. Standard infection-control measures—such as hand hygiene, the use of personal protective equipment (PPE), environmental cleaning and disinfection, and appropriate respiratory etiquette—should be consistently applied. Immunization, both active and passive, represents the cornerstone of prevention. Children with CHD should receive all routine childhood vaccines, as the risks associated with vaccine-preventable infections far outweigh potential adverse reactions. Although side effects such as fever-related tachycardia or transient cardiac decompensation may occur more frequently, they are generally manageable with antipyretics and short-term monitoring. Despite these considerations, vaccination coverage among children with CHD remains suboptimal [53,54,55]. Frequent illness-related delays, parental hesitation regarding vaccine safety and effectiveness, reluctance among some health-care providers to vaccinate children with underlying disease, and the absence of universally accepted CHD-specific immunization guidelines all contribute to low uptake [56,57].

The following sections summarize prevention strategies for major viral and bacterial pathogens relevant to CHD. The routine use of antibiotics as a prophylactic measure to prevent respiratory infections in children with CHD is not supported by strong evidence and should generally be avoided. Although antibiotics are sometimes prescribed empirically because of concern about infection-related decompensation in this vulnerable population, most respiratory infections in children with CHD are viral in origin, and inappropriate antibiotic exposure does not reduce infection incidence or severity. Conversely, unnecessary antibiotic use increases the risk of antimicrobial resistance, disrupts the developing microbiome, and may predispose patients to subsequent infections with multidrug-resistant organisms, particularly in those who require repeated hospitalizations or surgical interventions. Current guidelines therefore recommend reserving antibiotic therapy for situations with clear clinical, laboratory, or microbiological evidence of bacterial infection, and emphasize antimicrobial stewardship as a key component of infection prevention strategies in children with CHD.

### 5.1. Viral Infections

#### 5.1.1. Respiratory Syncytial Virus

Efforts to prevent RSV infection in children with CHD were historically hindered by the failure of early vaccines, particularly a formalin-inactivated preparation that caused vaccine-associated enhanced disease (VAED) upon exposure to natural RSV [58]. Progress was achieved with the introduction of palivizumab (PV) in the late 1990s. PV, a monoclonal antibody targeting a conserved epitope of the RSV F protein, demonstrated efficacy in high-risk infants, including those with hemodynamically significant CHD [59,60]. In a pivotal trial, PV reduced RSV-related hospitalizations by 45%, hospital days by 56%, and the need for supplemental oxygen by 73% in children ≤24 months of age [60], leading to broad recommendations for its use in CHD.

However, long-term experience revealed several limitations of PV, including modest overall efficacy, lack of impact on mortality, limited effect on post-RSV wheezing, poor adherence due to monthly dosing, and high cost [61,62]. Additional studies showed that continuing prophylaxis into the second year of life offered no meaningful benefit [62,63], and withholding PV from children aged 13–24 months did not adversely affect key clinical outcomes [64]. Consequently, PV recommendations were narrowed to CHD patients ≤12 months with significant hemodynamic compromise [65]. More recently, the American Academy of Pediatrics announced the retirement of its PV Technical Report, with PV expected to be withdrawn from the market at the end of 2025 [65,66].

RSV prevention has since been transformed by the arrival of two long-acting monoclonal antibodies—nirsevimab (NV) and clesrovimab (CL)—and by the introduction of RSV maternal vaccination. NV and CL target distinct conserved epitopes on the prefusion RSV F protein and have substantially longer half-lives than PV (NV ~71 days; CL ~45 days). A single injection provides five to six months of protection, ensuring coverage for the full RSV season and greatly improving compliance.

Among these, NV has been most extensively evaluated. Clinical trials demonstrate that a single NV dose prevents approximately 80% of medically attended RSV LRTIs and hospitalizations and about 85% of very severe RSV cases for up to 180 days [67,68]. Pharmacokinetic studies support extending its use to infants with severe underlying conditions, including CHD, both before the first RSV season [67] and with re-dosing prior to the second [69]. CL has also demonstrated strong efficacy in healthy infants—60.4% protection against medically attended RSV disease and 84.2% protection against hospitalization—but data beyond the first season are not yet available [70].

Current guidelines recommend universal NV or CL immunization for infants under eight months entering their first RSV season; for high-risk children entering their second season, only NV is recommended. However, CHD patients are not routinely included among high-risk older infants unless they have persistent hemodynamic instability or require continued therapeutic interventions [71]. Acceptance of NV has been high in countries where it has been incorporated into routine newborn immunization schedules, with up to 80% uptake [72], though coverage remains lower in low- and middle-income countries due to cost constraints.

Maternal RSV vaccination, using a stabilized prefusion F protein vaccine, offers another effective strategy. Vaccination between 24 and 36 weeks’ gestation reduces RSV-associated LRTIs by approximately 70% in infants up to six months of age, attributable to efficient placental antibody transfer [73]. This protection is expected to extend equally to infants with congenital heart defects identified postnatally.

Despite recent advances, currently available RSV prevention tools generally do not protect beyond six months of age. Notably, severe RSV disease still occurs in late infancy, underscoring the need for pediatric RSV vaccines that are presently in development [74].

#### 5.1.2. Influenza

Because children with CHD are at high risk of severe influenza, international health authorities recommend annual influenza vaccination for all patients with CHD, regardless of defect complexity [75]. This recommendation often extends to pediatric populations in countries where influenza vaccination is not routinely advised for healthy children [76].

Two vaccine types are available: inactivated influenza vaccines (IIV) and live attenuated influenza vaccines (LAIV). IIV mainly elicits serum IgG responses, while LAIV stimulates broader immunity across humoral, mucosal, and cellular pathways, more closely resembling natural infection. LAIV may therefore provide broader cross-protection, although its performance varies by circulating strains. Overall, both IIV and LAIV prevent at least half of influenza cases in children [77,78,79,80].

Despite strong evidence supporting vaccination, coverage among children with CHD remains inconsistent and often low, contrasting with generally higher uptake in adult populations [81]. Rates vary widely across countries: 65% in Greece [82], 64.6% in U.S. adults but only 36% in children [83], and as low as 6.6–8.6% among Belgian children [84]. Parental perceptions, provider recommendations, and interruptions caused by surgical treatment schedules all contribute to under-vaccination [83].

#### 5.1.3. SARS-CoV-2

Although in some regions non-mRNA COVID-19 vaccines were administered to children earlier in the pandemic, current international recommendations and regulatory approvals now support the use of mRNA vaccines only for pediatric populations [85,86,87,88]. Clinical trials and real-world data show that these vaccines substantially reduce severe COVID-19, hospitalization, multisystem inflammatory syndrome in children (MIS-C) [89], and long-term post-infectious complications [90]. Although vaccine effectiveness against infection is lower in younger children and varies with viral variants, protection against severe disease remains consistently high across age groups. Reported adverse events are usually mild and transient; myocarditis remains rare [86,87,88].

Vaccination policies for healthy children have differed widely across countries, influenced by perceived disease severity and hesitancy regarding mRNA technology [91]. For example, some nations, including Australia [92] and the United States [93], now leave vaccination of healthy children largely to parental discretion.

In contrast, there is a broad consensus that children aged ≥6 months with CHD should receive COVID-19 vaccination and appropriate boosters [92,93,94]. However, pediatric uptake remains low. In the U.S., only about 15% of children were up to date with COVID-19 vaccination in the 2024–2025 season, with just 5–6% coverage among those aged 6 months to 4 years [95]. In the UK, only 35% of children and adolescents received a first dose over a 10-month period, with even lower rates in younger age groups [96].

Few studies have specifically evaluated vaccine effects in children with CHD, but available data—mostly in adults—indicate substantial benefit. In a cohort of more than 57,000 vaccinated adults with CHD and COVID-19, vaccination reduced hospitalization from 15.8% to 0.5% and mortality from 4.6% to 0.5% [97]. Importantly, vaccination did not increase the baseline risk of inflammatory or thrombotic cardiac complications. Pediatric data also demonstrate strong protection: vaccination reduced COVID-19 hospitalizations by 87% in children with CHD [50]. Additional evidence shows that most children admitted to ICUs for COVID-19 after vaccine rollout were unvaccinated [98].

### 5.2. Bacterial Infections

Several bacterial infections relevant to CHD can be prevented through routine immunization, particularly with pneumococcal conjugate vaccines (PCVs) and *H. influenzae* type b (Hib) vaccine. The introduction of PCVs approximately 25 years ago dramatically reduced pneumococcal disease burden. Initial vaccines covered 7 serotypes, later expanded to 10 and 13 serotypes. Ongoing serotype replacement prompted the development of PCV15 and PCV20, both now authorized for pediatric use [99].

In high-income countries, PCVs are universally recommended beginning in early infancy, with either a two-dose primary series [100] or a three-dose schedule [101], followed by a booster at around 12 months. For children who receive PCV15, an additional booster with PCV20 or PPSV23 is recommended at least eight weeks after the final dose. Similar booster requirements apply to children vaccinated with PCV13 or those who received incomplete schedules [101].

Despite these recommendations, pneumococcal vaccination coverage among children with CHD often lags behind that of healthy peers. In one study from China, only 86.74% of children with CHD were fully vaccinated by 12 months, compared with over 90% coverage for routine vaccines [54]. Use of PPSV23 among eligible children was particularly low (10.2%) [102]. Factors contributing to low coverage include provider hesitancy, unclear guideline interpretation, limited clinical trial data specific to CHD, and parental concerns about vaccine safety [57].

Few studies have evaluated PCV effectiveness specifically in CHD populations. One analysis involving PCV7 and PCV13 demonstrated a significant reduction in all-cause pneumonia: vaccinated children had a 60.5% lower risk, with an absolute reduction of 20.3%, compared with unvaccinated peers [51]. The absence of differences by the number of doses received suggests that even partial vaccination confers meaningful protection. PCVs with broader serotype coverage are expected to maintain or enhance this benefit and should continue to be prioritized for children with CHD.

Table 3 summarizes the preventive immunization options for children with CHD, whereas Table 4 shows barriers to immunization and strategies to improve uptake.

## 6. Study Limitations

This review has several limitations that should be acknowledged. As a narrative review, the selection and synthesis of evidence were qualitative and did not include a formal systematic review methodology or quantitative meta-analysis, which may introduce a degree of subjectivity. Although a comprehensive search strategy was applied across multiple databases, the review was restricted primarily to publications available in English, which may have led to the exclusion of relevant studies published in other languages, including Spanish. Consequently, some regional data or locally relevant findings may not be fully represented. In addition, heterogeneity in study design, patient populations, CHD classifications, and outcome definitions limited direct comparisons across studies. Finally, high-quality evidence specific to children with congenital heart disease remains limited for several preventive interventions, requiring cautious interpretation and reliance on extrapolation from broader pediatric populations.

## 7. Conclusions

Children with CHD, particularly those with more severe forms, experience respiratory infections more frequently and with greater severity than their healthy peers. Effective preventive tools—including vaccines and monoclonal antibodies—are available for many of the most important viral and bacterial pathogens, and recent advances such as long-acting RSV monoclonal antibodies have markedly improved protection during early infancy. Despite these benefits, preventive interventions remain significantly underutilized, leaving many children with CHD unnecessarily vulnerable to infections that may lead to hospitalization, intensive care admission, or even death.

Underuse of these measures results from a combination of physician- and parent-related factors. Limited evidence specific to CHD populations has historically contributed to ambiguity in vaccination guidelines, reinforcing hesitation among pediatricians and fueling parental concerns about vaccine safety, efficacy, and potential adverse effects. Higher coverage is achievable when immunization strategies are universal, well accepted, and administered before CHD is diagnosed, as exemplified by the rollout of RSV monoclonal antibodies.

Improving protection for children with CHD will require a multifaceted approach. Priorities include strengthening clinical research to refine evidence-based immunization protocols; ensuring routine assessment of vaccination status during cardiology and specialty visits; implementing integrated systems that facilitate patient tracking and follow-up; enhancing primary care support through specialist collaboration; and providing parents with clear, consistent, and CHD-specific immunization guidance. Taken together, these strategies can meaningfully increase prevention coverage and reduce the burden of respiratory infections in this vulnerable population.

## Figures and Tables

**Table 1:** Immune Mechanisms Underlying Increased Infection Susceptibility in Children With Congenital Heart Disease.

Mechanism	Description	Associated CHD Types	Key References
Impaired neutrophil function	Reduced effector activity and bacterial killing	More common in severe CHD	[9]
Lymphocyte abnormalities	Reduced T and B cell counts; decreased naïve T-cell production	Critical, cyanotic, conotruncal defects	[10,13]
Reduced thymic output	Lower TRECs; thymus hypoplasia or surgical removal	Complex CHD; early surgery	[11,22,23]
Immunoglobulin deficiencies	Low IgA, IgG; reduced complement levels	Severe CHD	[12]
Chronic inflammation	Elevated IL-6, IL-10, TNF-α; abnormal hemodynamics; gut dysbiosis	Cyanotic CHD; VSD; coarctation	[14,15,16,17,18,19]
Hypoxia-related impairment	Reduced ROS and NETs formation	Cyanotic CHD	[20,21]
Genetic syndromes	Immune + cardiac defects in 22q11.2, DS, TS	Multiple CHD subtypes	[24,25,26,27,28,29,30,31]

CHD—Congenital heart disease; TRECs—T-cell receptor excision circles; ROS—Reactive oxygen species; NETs—Neutrophil extracellular traps; DS—Down syndrome; TS—Turner syndrome.

**Table 2:** Burden and Clinical Impact of Key Respiratory Viruses in Children With Congenital Heart Disease.

Virus	Major Clinical Findings in CHD	Comparative Risk vs. Healthy Children	Key References
RSV	Higher ICU admission, ventilation, mortality; worsened surgical outcomes	3–10× more severe disease	[32,33,34,35,36,37,38,39]
Influenza	↑ Mortality, respiratory failure, ventilation, AKI, LOS	2–3× higher risk of severe outcomes	[40,41,42,43]
SARS-CoV-2	↑ Arrhythmias, heart block, respiratory failure, AKI, ventilation	Higher morbidity; similar mortality	[44,45,46,47,48,49,50]

CHD—Congenital heart disease; RSV—Respiratory syncytial virus; ICU—Intensive care unit; AKI—Acute kidney injury; LOS—Length of stay.

**Table 3:** Preventive Immunization Options for Children with Congenital Heart Disease (Active and Passive).

Pathogen	Prevention Strategy	Age/Population	Effectiveness	Key References
RSV	Nirsevimab	Infants < 8 months; high-risk older infants	~80–85% reduction in severe RSV	[67,68,69]
RSV	Clesrovimab	Infants < 8 months	60–84% protection	[70]
RSV	Maternal vaccine	24–36 weeks of gestation	~70% protection for 6 months	[73]
Influenza	IIV/LAIV annual vaccination	All CHD children ≥ 6 months	≥50% effectiveness	[75,76,77,78,79,80,81,82,83,84]
SARS-CoV-2	mRNA vaccines	≥6 months; high-risk groups	Strong protection vs. severe disease	[85,86,87,88,89,90,91,92,93,94,95,96,97,98]
*Streptococcus pneumoniae*	PCV13/15/20 + PPSV23	Standard pediatric schedule	~60% pneumonia reduction	[51,54,57,99,100,101,102]
Hib	Hib vaccine	Standard schedule	High effectiveness	—

RSV—Respiratory syncytial virus; IIV—Inactivated influenza vaccine; LAIV—Live attenuated influenza vaccine; CHD—Congenital heart disease; PCV—Pneumococcal conjugate vaccine; PPSV23—23-valent pneumococcal polysaccharide vaccine; Hib—*Haemophilus influenzae* type b.

**Table 4:** Barriers to Immunization and Strategies to Improve Uptake in Children with Congenital Heart Disease.

Barrier	Description	Proposed Strategy
Delayed vaccination	Frequent illness/hospitalization postpones doses	Check immunization status at every cardiology visit
Provider hesitancy	Unclear guidelines and safety concerns	Update protocols; specialist collaboration
Parental concerns	Safety, efficacy, and side-effect worries	Provide CHD-specific counseling and guidance
Limited evidence	Few CHD-specific vaccine trials	Support further clinical research
Poor care coordination	Fragmented follow-up between specialists and primary care	Use integrated tracking systems
Cost/availability	High costs limit mAb uptake in LMICs	Broaden access and reimbursement programs

CHD—Congenital heart disease; LMICs—Low- and middle-income countries; mAb, monoclonal antibody.

## Data Availability

Not applicable.
